# Bovine Lymphocyte Intestinal Retention Defect (BLIRD): a novel recessive immunogenetic disorder in Holstein cattle

**DOI:** 10.1080/01652176.2025.2566997

**Published:** 2025-10-15

**Authors:** Lucie Dutheil, Blandine Gausseres, Florian Besnard, Laurence Guzylack-Piriou, Yanad Abou Monsef, Nicolas Gaide, Lisa Arnalot, Fabien Corbiere, Marie Gaborit, Frédéric Launay, Agnès Poujade, Aurélien Capitan, Gilles Foucras

**Affiliations:** a Univ Toulouse, ENVT, INRAE, IHAP, Toulouse, France; b Institut de l’élevage, Paris, France; c Univ Paris-Saclay, INRAE, AgroParisTech, GABI, Jouy-en-Josas, France; d Animal Sciences, Ecole d’Ingénieurs de Purpan, Toulouse, France; e Univ Toulouse, ENVT, INP, INRAE, GenPhySE, Toulouse, France; f Unité Expérimentale du Pin, INRAE, UE326, Le-Pin-au-Haras, France

**Keywords:** Integrin β7, BLIRD, immune disorder, leucocytosis, lymphocyte, eosinophil, intestine

## Abstract

Dozens of missed recessive loci affecting homozygous carriers’ life expectancy were recently reported. This article details the clinical, biological and pathological manifestations of a new bovine genetic disorder caused by the ITGB7 p.G375S point mutation in the French Holstein cattle breed (BLIRD: OMIA:002872–9913). Our thorough study involved database analysis of genotyped cattle and a series of case-control investigations of forty individuals homozygous for the causative variant. These variant homozygotes had a significantly shorter lifespan (fewer than 64% surviving past three years vs. 87% in control), along with reduced body weight, daily weight gain, and dairy performance. The mutation did not affect most biochemical parameters, but a marked lymphocytic leucocytosis, moderate eosinophilia and differences in faecal microbiota were observed. Although non-pathognomonic symptoms may be confused with those of common environmental diseases, the blood profile effectively identified suspected carriers who developed ill-thrift and poor growth as heifers. Our research demonstrates that the bovine ITGB7 p.G375S substitution leads to reduced longevity, poor condition and production in most homozygous carriers. Furthermore, this spontaneous model may help to refine the functions of the integrin β7 (ITGB7) in immune homeostasis and defence.

## Introduction

Genetic selection (GS) has played a crucial role in advancing dairy cattle breeding, particularly in enhancing milk production, by the global use of a limited number of common dairy breeds such as Holstein and selected sires over the past century (Wiggans and Carrillo 2022). This strategy has contributed to significant improvements in the genetic potential of dairy cattle. Additionally, the application of genomic information in bull breeding has enabled the selection of desired traits more effectively, while the integration of single nucleotide polymorphism (SNP) data has enhanced breeding efficiency by providing more precise predictions of genetic merit. While these advancements have led to remarkable gains in productivity, they have also raised concerns about the erosion of genetic diversity within dairy cattle populations. This reduction could undermine disease resistance and potentially shorten the lifespan of dairy cattle. Despite these breeding advances, the average lifespan of dairy cattle remains relatively short, typically between 4.5 and 5.5 years, with a productive life span of only 2.5 to 3.5 lactations, which is well below their expected life expectancy (Schuster et al. 2020; Dallago et al. 2021). Approximately 20% of dairy heifers do not survive to their first calving (Zhang et al. 2019; De Vries and Marcondes 2020), and early culling, driven by factors like health problems and reproductive failure, leads to significant economic losses (De Vries 2020). The narrowing of genetic diversity within the Holstein breed has further compounded these concerns. As of 2017, the average genomic inbreeding level among Holstein bulls in the United States was 12.7%, with cows showing an inbreeding level of 7.9% (Guinan et al. 2023). This inbreeding has negative effects not only on the longevity of dairy cattle but also on their production, fertility, and health traits (Mugambe et al. 2024). Moreover, the prevalence of genetic disorders is a growing concern, with early studies (Robertson 1970; Charlesworth and Charlesworth 1999), laying the groundwork for understanding the genetic basis of many inherited conditions. More recent studies (Daetwyler et al. 2007) highlight the impact of modern breeding practices on the spread of genetic disorders.

In addition to outstanding progress in animal breeding and dairy production through the identification of preferred traits (Brito et al. 2021) and the prediction of genetic merit, recent advances in genomic technologies, such as genome-wide association studies (GWAS) and whole-genome sequencing, have greatly enhanced the detection of recessive mutations associated with impaired fitness in livestock. These include mutations causing embryonic lethality, such as Holstein haplotypes HH1 and HH2 (Shanks et al. 1984; Agerholm et al. 2006; Charlier et al. 2012; Häfliger 2022); those leading to postnatal pathological phenotypes, such as retinal degeneration (Häfliger et al. 2022); as well as variants associated with juvenile or late-onset mortality. These deleterious alleles, typically identified through positional cloning and haplotype-based mapping approaches, underscore the unintended consequences of intense selection and the recurrent use of a limited number of sires (Shuster et al. 1992; Fritz et al. 2013).

Building on these approaches, Besnard et al. (Besnard et al. 2024) recently applied a method based on haplotype enrichment and depletion in cohorts of animals with distinct life histories, leading to the identification of 13 novel recessive loci in Holstein cattle associated with juvenile and late mortality. Among them, several haplotypes reach population frequencies above 2.5% in the French Holstein genotyped population, including H11P34, H17P66, H14P1, H29P38, H11P78, H4P71, and H2P34.

One particularly notable discovery is called « Bovine Lymphocyte Intestinal Retention Defect » (BLIRD), which is caused by a substitution of Glycine to Serine at amino acid position 375 in bovine integrin β7 (ITGB7_ AAI34677.1:p.(G375S)). This mutation has an allele frequency of 4.8% in the French Holstein population, with approximately 2.3 homozygotes per 1,000 individuals (Besnard et al. 2024). BLIRD, catalogued in OMIA (entry 002872–9913 OMIA:002872–9913, https://omia.org/OMIA002872/9913/) (Nicholas and Tammen, 1995), has been reported not only in France but also in Australia (Van Den Berg et al. 2024) Switzerland (Leuenberger et al. 2024) and the USA (Al-Khudhair et al. 2025), highlighting its global relevance and emphasising the significance of worldwide awareness for veterinary diagnosis, herd management, and genetic counselling.

This deleterious substitution affects a residue that is completely conserved across 128 vertebrate orthologs encoding for integrin β7, a member of a family of adhesion molecules. The protein forms a heterodimer with integrin α4 to create the α4β7 complex (also known as LPAM-1), a key cell adhesion molecule expressed predominantly on activated or memory CD4+ T lymphocytes (Rüegg et al. 1992). This heterodimer plays a pivotal role in the trafficking of T cells from the bloodstream to the gastrointestinal tract (Gorfu et al. 2009), which is essential for maintaining intestinal immunity (Berlin et al. 1993). α4β7 is also constitutively expressed on naïve T and B cells at a relatively low level. Its expression is tightly controlled during lymphocyte activation and differentiation into effector and memory T cells (Cimbro et al. 2012). It is also expressed in Natural Killer (NK) cells, stimulated monocytes, macrophages and eosinophils (Gorfu et al. 2009). Integrin β7 is shared by several subtypes of leukocytes including lymphocytes (Andrew et al. 1996; Keir et al. 2021), non-classical monocytes (Schleier et al. 2020) and eosinophils (Walsh et al., 1996). The α4β7 complex is believed to be gut-specific due to its binding to Mucosal Addressin Cell Adhesion Molecule 1 (MAdCAM-1), which is exclusively expressed on intestinal endothelial cells and facilitates leukocyte extravasation into intestinal high endothelial venules (Shouval 2022). As suggested by preliminary pathophysiological analyses on a small cohort of cases and control Holstein cattle (Besnard et al. 2024) and data from the literature (Wagner, 1996), the replacement of a glycine with a neutral serine may affect integrin β7’s functions thereby disrupting the homing of immune cells to the gastrointestinal tract, and the maintenance of mucosal integrity. From a pathological perspective, the dysregulation of integrin β7 may contribute to the development of inflammatory diseases, characterised by aberrant immune cell trafficking to the gut, which is partly mediated by the α4β7 dimer (Feagan et al. 2013). In view of these observations, the objective of our current study is to investigate the phenotypic, lesional, and immunological consequences of BLIRD in Holstein cattle. By conducting comprehensive analyses of large genotype and phenotype databases, on-field clinical examinations, necropsies, and laboratory tests, we present novel data elucidating the immune consequences and underlying pathophysiology of BLIRD.

## Materials and methods

### Outline of the participant’s attributes and equipment description

Blood samples were collected by licenced veterinarians, with owner consent for diagnostic testing. No animal was purposely bred for the study, and invasive sampling was performed post-mortem. All data were obtained with permission from French breeders and breeding organisations (Auriva, Synetics, Genes Diffusion)**.**

### Animals and records available from the French National Bovine Database

This study takes advantage of the large amount of genetic and phenotypic information recorded for management and selection purposes in the French Holstein population. We extracted from the French National Bovine Database the genotypes for the BLIRD for a total of 740,276 females genotyped at a young age (typically within their first three months of life) with successive versions of the Illumina EuroGMD custom SNP array from 2019 to date as part of routine genomic evaluation (for details on the probe used, see Besnard et al. (Besnard et al. 2024). We also extracted a set of information on various life events (dates of birth, death, artificial insemination (AI), calving, lactation start and end, and cause of death), as well as yield deviations corrected for environmental effects, computed within the framework of genomic evaluations for the females that started a productive career. This information allowed us to create the appropriate cohorts for each analysis performed in this study (see Supplementary Table 1 for details on traits and cohort sizes).

### Population-level survival analysis

The effect of BLIRD on juvenile mortality and premature culling was studied over a period of three years, corresponding to two years of rearing and one year of productive life in conventional French Holstein dairy farms. A total of 203,180 homozygous reference (ref/ref - BLIRD free - LRF) 21,746 heterozygous (ref/var - BLIRD carrier – LRC), and 437 homozygous variants (var/var – BLIRD affected – LRS) born at least three years before the analysis date, were selected, and over the period considered, we calculated the daily counts and proportions of animals alive, dead by euthanasia or natural causes, or slaughtered for each genotype. Finally, at the end of the period, the proportion of live animals was compared pairwise between genotypes using Fisher’s exact test on the number of live animals versus the sum of dead and slaughtered animals (see Supplementary Table 2 for details).

### Population-level analysis of performance records

The effects of homozygosity and heterozygosity for the ITGB7 p.G375S substitution were estimated on 14 production, morphological and fertility traits routinely recorded for selection purposes. Yield deviation data, i.e. records adjusted for the non-genetic effects included in the models used for the official genomic evaluations carried out on behalf of the French breeding organisations were obtained from GenEval (for details on the models used, see https://www.geneval.fr/english) for cohorts of 38,751 to 247,206 LRF, 3723 to 25,698 LRC, and 36 to 262 LRS females, depending on the trait.

A mixed animal model including the fixed effects of the substitution (0 versus one or two copies) and year of recording, along with the individual random polygenic effect, was used to estimate the effects without bias. Calculations were performed using blupf90+ from the *BLUPF90* suite of programs (Misztal et al. 2014). To assess statistically significant differences between the group means of each trait, a Student t-test was applied using the stats R package. P-values were adjusted using the Benjamini-Hochberg method to control the false discovery rate (Benjamini and Hochberg 1995). Finally, effects were standardised to genetic standard deviations (GSD) based on genetic parameters estimated from national genomic evaluations, to facilitate trait comparison.

In addition to yield deviations, we analysed raw performances for three traits that are not considered in genomic evaluations but that we considered relevant for this study: age at first AI, which can be used as a proxy for growth in the absence of specific recording (Jourdain et al. 2023), age at first calving, which is highly correlated with the previous one, and duration of the first lactation. The means between each genotype group were compared using a Student’s t-test.

### Retrospective analysis of heifer growth in an experimental farm

Monthly weight data recorded during the first two years of life were obtained from the INRAE experimental unit at Le-Pin-au-Haras (Orne, France) for 719 LRF, 131 LRC and 4 LRS. The growth curves of each animal were determined using regression. Then, the analyses performed included the determination of the curves corresponding to the 10th and 90th percentile and the mean curves for the LRF and LRC. Due to the small number of LRS animal and the fact that they all died before reaching the end of the rearing period, individual growth curves were displayed for this group.

### Field survey

A field survey was conducted to evaluate further the point mutation impact drawn from population records. From a list of 314 alive LRS distributed throughout France, 40 were selected in 38 commercial herds distributed across Normandy and the Southwest region of France. The study’s methodology involved the choice of a matched control in the same herd with the lowest age difference, in most cases less than one month. Following prevailing farm practices, the animals commonly received preventive treatments, including respiratory vaccines, deworming drugs, and anticoccidials. However, these treatments were not the subject of evaluation in the present study. In total, 86 animals were included in the survey and subjected to a close clinical examination (40 LRS,10 LRC and 36 LRF). Each animal was clinically examined and photographed alongside its control. Live weight was indirectly assessed through the thoracic circumference using a barometric ribbon, and body weight was deduced using the Crevat formula (P = K T^3^, where T is the chest circumference in metres, K is a coefficient that has been calculated on average at 80). Three of the homozygotes included in the field survey were brought to the ruminant clinic of the National Veterinary School of Toulouse (ENVT) for detailed clinical examination and tissue sampling after necropsy.

### Laboratory analysis

Blood samples were collected from the 86 animals of the field survey for subsequent serum analysis and complete blood count. Venous blood sampling was conducted in BD Vacutainer UU Plastic 4 ml blood collection tube EDTA K2, Lithium Heparin or dry with activator (BD Biosciences, France). The blood samples were stored at 4 °C, and processed within 12h by centrifugation for serum and plasma separation. Samples collected in EDTA tubes were used for haematological purposes, as heparin and dry tubes were used for biochemistry and serology, respectively.

#### Biochemistry

For biochemistry, the following parameters were assessed on heparin anticoagulated plasma: total proteins, albumin, urea, and aspartate aminotransferase (ASAT) in 54 animals (27 LRS and 27 LRF/LRC). Globulins were calculated as the difference between total protein and albumin. Analyses were conducted using a Vitros 250 analyser and appropriate multi-layer reagents (Ortho Clinical Diagnostics, Issy les Moulineaux, France), following the manufacturer’s instructions. Quality control of the Vitros 250 was performed using the Performance Verifier I & II solutions (Ortho Clinical Diagnostics, Issy les Moulineaux, France).

#### Complete blood count

Prior to analysis, the EDTA blood tubes were held at room temperature for 20 min, during which time they were gently agitated to ensure homogenisation. Measurements were conducted within a 12-hour window after sampling, carried out by the CREFRE - Inserm - UPS - ENVT Comparative Medical Biology and Histology platform using Sysmex XN-V and Sysmex XT-2000iV software. Blood cell enumeration was also done using air-dried blood smears and staining with a May-Grünwald/Giemsa automatic Stainer. A comprehensive range of variables was analysed, including impedance and optical red blood cell (RBC) counts, haematocrit, Haemoglobin concentration, mean corpuscular volume, red cell distribution, platelet counts, mean platelet volume, platelet crit, platelet distribution width, and platelet large cell ratio. The variables measured by the analyser include the white blood cell (WBC) count, as well as neutrophil, lymphocyte, monocyte, eosinophil and basophil counts. These were determined from 100 leukocytes counted per oil immersion field (Grebert et al. 2021).

### Flow cytometry analysis

Flow cytometry was conducted on mononuclear cell fractions from blood, mesenteric lymph node (mLN) and lamina propria (LP) of LRS (*n* = 3) and LRF/LRC controls (*n* = 4). PBMC were prepared from EDTA-anticoagulated whole blood diluted 1:1 with phosphate-buffered saline (Dutsher, Cat#L0615), layered on half the volume of Ficoll Paque Plus (Cytiva, Cat#17-1440-03) and centrifugated at 1200 xg for 20 min at room temperature with the brake off. Red blood cells were lysed with ACK lysis buffer (154 mM ammonium chloride 10 mM potassium bicarbonate, 97 µM EDTA, pH 7,4). mLN were smashed using a syringe piston and a 70 µm cell strainer to produce a cell suspension.

The small intestinal (jejunum) wall was washed in cold PBS, cut into pieces, and incubated four times with 3 mM EDTA for 40 min at 37 °C. Tissue pieces were then rinsed and digested in DMEM with 20% FCS and 100 U/ml of collagenase for 40 min further at 37 °C. The suspension was filtered and undigested pieces were smashed on a 70 µm cell strainer using a syringe piston. Finally, mononuclear cells were isolated using a 40–80% Percoll gradient.

Cells were resuspended in HBSS with 0.5% BSA and 10 mM Hepes. Cell viability was assessed using Viobility 405/520 Fixable Dye (Miltenyi Biotec, Cat#130-130-404). The antibodies used were: CD45 FITC (BioRad, Cat#MCA2220), CD4 Pacific Blue (BioRad, Cat#MCA1653), CD45RO Alexa Fluor 647 (BioRad, Cat#MCA2434), α4 integrin PE/Cyanine7 (BioLegend, Cat#304314) and integrin β7 PE (BioLegend, Cat#121006).

Antibody incubation was performed for 20 min at 4 °C in the dark. Data were collected on a MACSQuant^®^ Analyser (Miltenyi Biotec) and subsequently analysed using FlowJo software (Becton Dickinson).

### Faecal microbiota composition

To assess the composition of the gut microbiota, faeces were collected directly from the rectum and placed in a leak-proof plastic container. Bacterial microbiota from 42 samples (21 LRS and 21 LRF/LRC) was investigated as described by Arnalot et al. (Arnalot et al. 2025). DNA was extracted from faecal samples using the Quick-DNA Faecal/Soil Microbe Miniprep Kit (Zymo Research, Irvine, CA, United States of America) according to the manufacturer’s instructions. The 16S rRNA V3-V4 region was investigated, and the sequencing was performed using MiSeq Illumina Sequencing at the Genomic and Transcriptomic Platform (INRAE, Toulouse, France). The sequenced reads were analysed using FROGS v4.1.0 software (Escudié et al. 2018). Briefly, 1,709,057 raw sequences were pre-processed, resulting in 1,492,612 sequences retained (87.3%), with the number of reads per sample ranging from 23,552 to 39,187. A total of 2,173 clusters and 1,196,762 sequences were retained, with a range of 18,456 to 32,470 sequences per sample. The subsequent data were computed in a *phyloseq* object for further R analysis (McMurdie and Holmes 2013).

### Serological testing and pathogen detection

Both serological assays and pathogen detection through polymerase chain reactions (PCR) were employed on 27 couples in the field study cohort. Diagnostic tests were performed using commercially available kits from ID vet Genetics and ID Screen, following the manufacturer’s protocols. A duplex PCR assay (ID Gene Paratuberculosis Duplex, ID vet Genetics) was used to detect *Mycobacterium avium* subsp. *paratuberculosis* (Johne’s disease) on DNA extracted from faecal samples. For the concurrent detection of Bovine Viral Diarrhoea Virus (BVDV), a triplex PCR assay (ID Gene BVDV/BDV Triplex 2.0, ID vet Genetics) was used, with blood-extracted RNA serving as the test matrix. In addition, serological screening for Bovine herpesvirus I and BVDV antibodies was conducted using enzyme-linked immunosorbent assays (ELISA), with ID Screen IBR gB Competition (Innovative Diagnostics, Grabels, France) to detect antibodies against the gB Bovine Herpesvirus type 1 (BoHV-1) glycoprotein and ID Screen BVD p80 Antibody Competition (Innovative Diagnostics, Grabels, France) for BVDV against the NS3 protein, with serum as the matrix. For Paratuberculosis, an indirect ELISA (ID Screen Paratuberculosis Indirect) was also used to detect antibodies against *M. avium* subsp. *paratuberculosis* in bovine serum. qPCR analysis was done on DNA faecal extracts to quantify the presence of *Ostertagia/Cooperia* eggs in the faeces of select animals (Baltrušis et al. 2019). All diagnostic assays were conducted in strict accordance with the manufacturer’s instructions.

### Post-mortem assessment & histopathology

A complete necropsy was performed after euthanasia on three LRS heifers, 28 months-old (*n* = 2) and 17 months-old (*n* = 1). For histopathology section of heart, liver, spleen, thymus, lung, rumen, abomasum, duodenum, jejunum, ileum, caecum, proximal and spiral colon, rectum, laryngeal and lingual tonsils, as well as peripheral and loco-regional visceral lymph nodes (left ruminal, ventral abomasal, jejunal, caecal, cranial mesenteric and caudal mesenteric, left mandibular, superficial cervical, subiliac, deep popliteal, iliofemoral, left tracheobronchial) were collected. All samples were fixed in 10% neutral buffered formalin for histopathological examination and immunohistochemistry. In parallel, intestinal sections (ileum) and the draining lymph node from five LRF/LRC Holstein female cattle were collected at the slaughterhouse to provide a histological comparison of tissues within normal limits.

Fixed tissue samples were paraffin-embedded and cut at 3 µm. Sections were stained with haematoxylin and eosin (H&E) for histopathological analysis. Immunohistochemistry (IHC) was performed to determine the distribution and abundance of lymphocytic B and T populations in the jejunum, jejunal lymph node and prescapular lymph node tissues, using CD3 (T-lymphoid cell marker, mouse monoclonal, Clone F7.2.38, Agilent, 1/50, M 7254) and CD20 (B-lymphoid cell marker, rabbit polyclonal, Thermo scientific, 1/600, PA5-16701). Briefly, the IHC protocol included an antigen retrieval step with buffer solution (EnVision Flex Target Retrieval Solution, Low and High pH for CD3 and CD20 respectively, Agilent) applied for 30 min at 96 °C, a peroxidase blocking step of 5 min at room temperature (S2023; Agilent) followed by saturation of non-specific binding sites with normal goat serum (X0907; Agilent) applied for 20 min at room temperature, and primary incubation for 50 min at room temperature. Signal amplification and revelation were assessed using the EnVision FLEX system and 3,3-diaminobenzidine (DAB) revelation according to the manufacturer’s recommendations.

### Statistical analysis of the data from the field survey cohort

Data distribution was assessed using the Shapiro–Wilk test. For comparisons between two independent groups, normally distributed data were analysed using a Student’s t-test, while non-normally distributed data were analysed using a Mann–Whitney U test. The 10 LRC and 36 LRF were considered suitable and merged into a single LRF/LRC group due to no notable differences (Mann-Witney, p-value < 0.05), in any of the examined traits. All the results are provided in Supplementary Table 2.

Data visualisation and statistical analysis were performed using R software (R Core Team 2023).

Generalised linear mixed models (GLMMs) were used for multivariate analysis, with response variables comprising morphological or biological parameters, including chest circumference, Haemoglobin levels, lymphocyte count, monocyte count, eosinophil count, basophil count, urea levels, globulin levels and aspartate transaminase levels. Explanatory variables included the Genotype, Age and Farm. The *glmmTMB* package (Brooks et al. 2017) was utilised to fit a series of models with varying degrees of complexity. The study considered various models, including simple models with fixed effects of Genotype and Age. The investigation also included the implementation of more intricate models that utilised natural B-splines to ensure the smoothness of the relationship between the quantitative variables and the outcome. The examination involved the interaction between Genotype and Age, models with or without random Farm effects, and where appropriate, models incorporating random farm effects and an interaction between genotype and age, as well as potential interactions between all three factors. The set of candidate models was fitted for each response variable, exploring various combinations of fixed and random effects. The most suitable model was selected based on the Akaike Information Criterion (AIC). The *emmeans* (Emmeans 2024) package was used to calculate estimate the marginal effects of genotype at different ages, with Age in models that included it as a fixed factor in the models. Marginal estimates were calculated at different ages and compared between Genotypes.

Multivariate relationships among continuous variables were examined using multiple factor analysis (MFA) and principal component analysis (PCA). Hierarchical clustering on principal components (HCPC) was then performed to assess genotype robustness. MFA, PCA, and HCPC were performed using the *FactoMineR* package (Lê et al. 2008).

Partial least squares discriminant analysis (PLS-DA) and sparse partial least squares discriminant analysis (sPLS-DA) were used to improve the ability of the model to discriminate linear combinations of variables and to identify the most influential variables in genotype classification *via* the *MixOmics* package (Rohart et al. 2017). This supervised method reduces the dimensionality of the data and has been shown to provide superior results in terms of improved genotype separation. This technique helps to discriminate between two sets of labelled points by identifying the most influential variables in genotype classification.

Regarding faecal bacterial community, *adonis2* from the *vegan* package (Oksanen et al. 2025) was used to test for β-diversity significance between groups. The significant differential taxa were tested with Analysis of Compositions of Microbiomes with Bias Correction 2 (*ancom-bc2,* package *ANCOMBC (*Lin et al. 2022; Lin and Peddada 2020)).

Statistical significance was defined as a p-value less than 0.05, and the results meeting this criterion were considered significant.

## Results

### Retrospective analyses indicate that BLIRD point mutation is associated with reduced longevity and poor zootechnical performance

**Figure 1.**
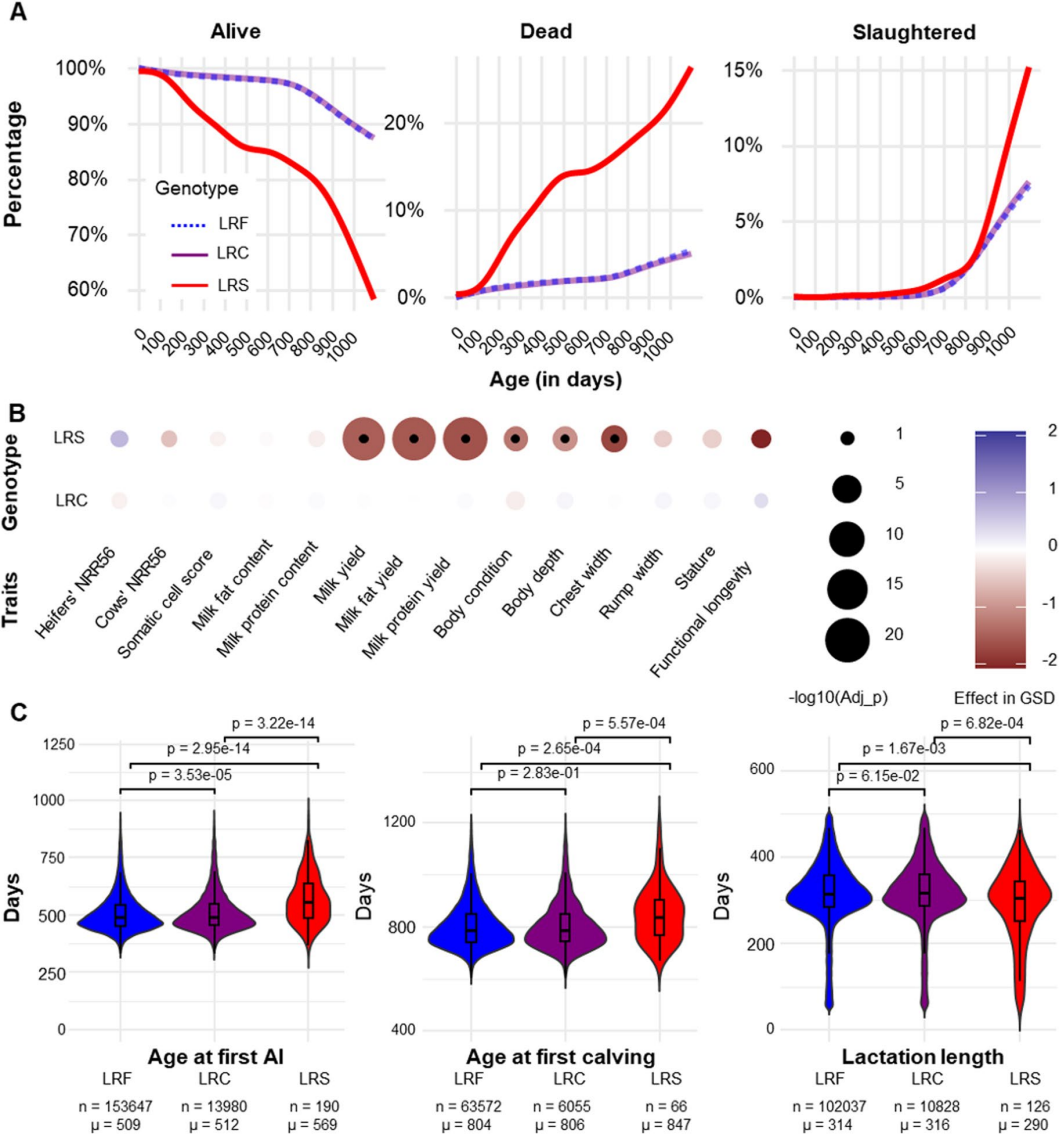
Population scale analyses of survival and performance records for holstein females genotyped for the ITGB7 p.G375S substitution. (A) Proportion of animals alive, dead (by euthanasia or natural causes), or slaughtered for 203,180 LRF, 21,746 LRC, and 437 LRS animals over three years, corresponding to two years of rearing and one year of productive life in conventional French Holstein dairy farms. (B) Effects of homozygosity and heterozygosity for the ITGB7 p.G375S substitution on 14 traits expressed in genetic standard deviation (GSD; see Supplementary Table 4 for details on cohort sizes). -log10(Adj-p): -log10 of Benjamini–Hochberg adjusted Student’s t-test p values. (C) Analysis of age at first AI, age at first calving and duration of first lactation. Means and cohort sizes are shown below the violin plots for each genotype group. The p-values shown for each comparison were obtained using a Tuckey test.

To gain insight into the consequences of the BLIRD on bovine health and longevity, we first studied the survival over three years of a large cohort of genotyped females (*n* = 203,180 LRF, 21,746 LRC and 437 LRS) for the corresponding DNA variant as part of the routine genomic evaluation. Survival, mortality and slaughter curves are shown in Figure 1A.

The red curve corresponds to LRS; meanwhile, the blue and purple curves correspond to LRF and LRC respectively. The curves for these two categories overlap. On the other hand, LRS showed a significant reduction in the proportion of live animals at the end of the period with only 63.6% versus 87.4% for the LRF and 87.5% for the LRC (Fisher’s exact test p-values =7.90 × 10^–37^ and 3.13 × 10^–36^ for each comparison respectively; Supplementary Table 3). This difference was due to a continuous excess due to either natural death and/or early slaughter between 2.5 and 3 years.

To investigate the causes of this increase in premature culling, we analysed yield deviations (i.e. phenotypes adjusted for environmental effects) for 14 traits considered in the French genetic evaluations, using a mixed model including the fixed effect of the ITGB7 substitution (0 versus 1 or 2 copies), the fixed effect of the year of recording, and the individual random polygenic effect (Supplementary Table 4 and Figure 1B). The LRS appeared to have normal fertility, as measured in heifers (nulliparous) and cows (multiparous) using the 56-day nonreturn rate (NRR56, which considers the proportion of females that have not been re-inseminated 56 days after previous insemination and are considered pregnant). However, those showed significantly lower performance in several production and morphological traits. The LRS status had no effect on milk composition (i.e. fat and protein content) but a strong negative effect on quantity, reducing milk yield by 1359 kg (equivalent to −1.79 genetic standard deviation or GSD; Benjamini Hochberg adjusted Student’s t-test p-value (FDR) =3.30 × 10^–22^), milk fat yield by 58.02 kg (−1.86 GSD, FDR =4.57 × 10^–24^) and milk protein yield by 43.87 kg (−1.93 GSD, FDR =5.87 × 10^–24^). Highly negative significant effects in homozygotes were also observed (Figure 1B) for body condition (−1.50 GSD, FDR =6.74 × 10^−4^) body depth (−1.21 GSD, FDR =8.01 × 10^−4^) and chest width (−2.07 GSD, FDR =4.74 × 10^−5^). Of note, suggestive but not significative effects after accounting for multiple testing were also observed in LRS for stature (−0.51 GSD, raw p-value (raw-p) =0.04) and functional longevity (−2.45 GSD; raw-p-value = 0.02). Therefore, the available yield deviations are those less affected by the BLIRD. To further investigate the effects of BLIRD on larger and less censored populations we studied raw phenotypes for three traits (Figure 1C). Age at the first insemination was significantly delayed by approximately 2 months in the LRS compared to the other genotypes (mean = 569 versus 512 and 509 days for the LRC and LRF, respectively; Tukey test p-value = 3.22 × 10^–14^ and 2.95 × 10^–14^ for the comparisons with each group respectively). Similar results were obtained for the age at first calving which is highly correlated to the latter trait in the absence of fertility problems. Finally, in concordance with the higher rates of premature culling observed in the survival analysis, the duration of the first lactation was also reduced by three to four weeks on average in the LRS as compared with the other genotypes (mean =290 versus 316 and 314 days for LRC and LRF, respectively; Tukey test p-value = 6.82 × 10^−4^ and 1.67 × 10^−3^ for the comparisons with each group respectively).

**Figure 2.**
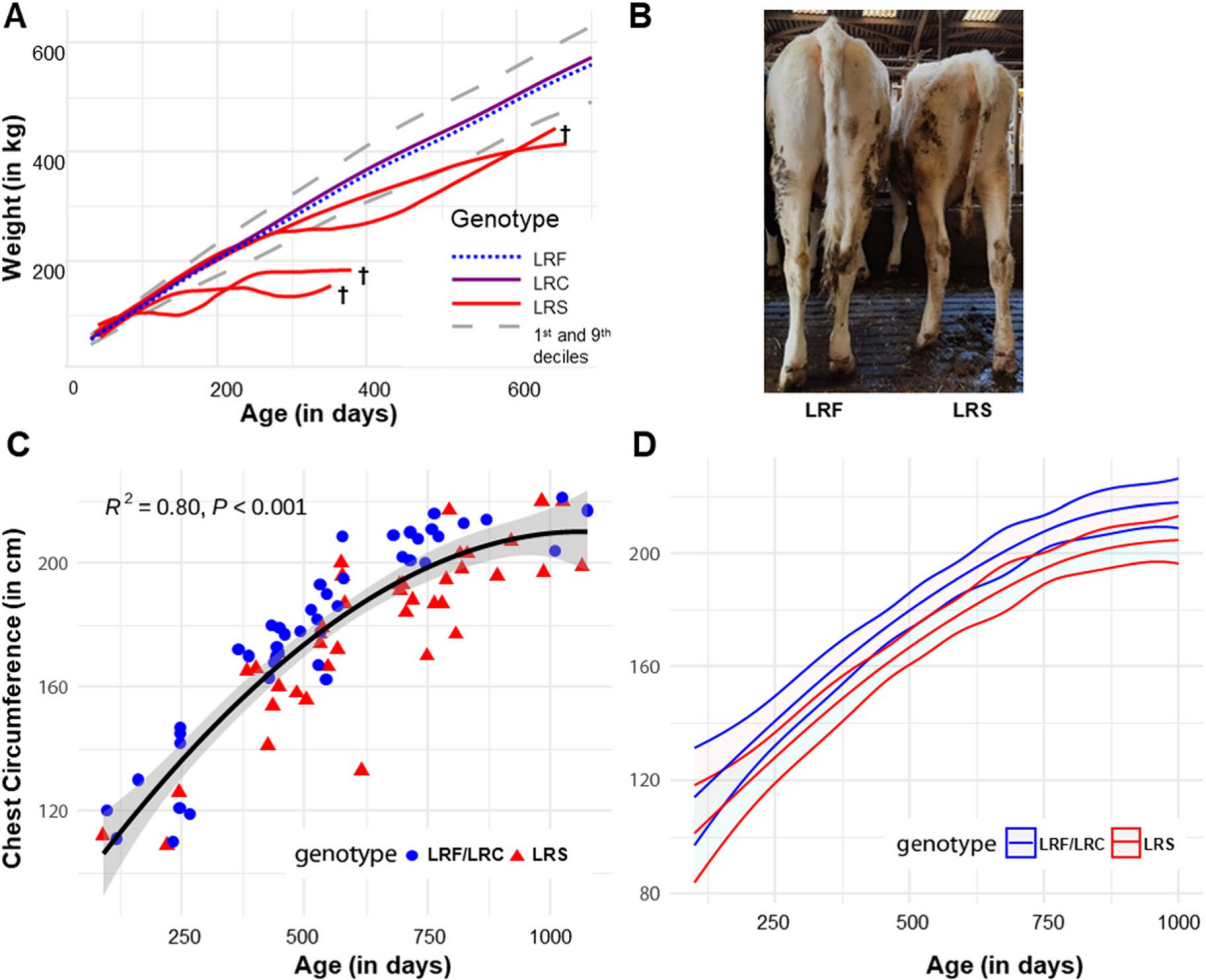
Growth data of ITGB7 p.G375S LRS (*n* = 40) and LRF/LRC(*n* = 46). (A) Weight growth curves for LRC, and LRS animals over 0–730 days in a single experimental herd. The graph presents the average weight curves for each genotype (LRF, LRC, and LRS), along with the 10th and 90th percentiles derived from the total dataset. Individual weight curves are also displayed for the 4 LRS. (B) The ill-thriving phenotype of a LRS and its age-matched LRF control, with less than 1-month difference. (C) Measured chest circumference (in cm) across age (days) for LRS (red triangle) and LRF/LRC (blue round) animals. (D) Modelled impact of the ITGB7 p.G375S substitution on chest circumference over time according to the LRS or LRF/LRC status.

To further investigate this effect on heifer growth, we analysed the monthly weight data of 719 LRF, 131 LRC and 4 LRS heifers routinely recorded during their first two years at the INRAE experimental unit located at Le-Pin-au-Haras (Orne, France). The results showed (Figure 2A) that the growth curves of the LRC (purple curve) individual were not different from those of animals of the LRF (blue curve). However, the four LRS (in red) exhibited a growth curve that consistently fell below the lower tenth percentile of the farm’s growth curves during the period considered. Moreover, three cases died prematurely at 351, 368 and 653 days of age without prodromal signs.

### Growth retardation and ill-thrift in BLIRD homozygote carriers on-field

According to the results of the clinical examination on farms, many cases displayed reduced stature, sparse coats, and occasionally a larger abdomen. Despite exhibiting signs of ill thrift, no diarrhoea or loss of appetite was reported. Figure 2B depicts one LRS and its matching control (LRF). The average chest circumferences and deduced weight of the LRS were not significantly different from those of the LRF/LRC (178 ± 27 VS. 178 ± 31 cm, Mann-Whitney test, p-value = 0.74). However, a graphical representation of chest circumference (in cm) according to the age (in days) (Figure 2C) reveals two discernible clusters. A pattern emerges in which animals carrying the mutation fall below the third-degree polynomial regression curve of the Holstein breed standards (Smith 2007), whereas LRF/LRC tend to be above or on the curve, regardless of their age at measurement. Notably, the LRS group is significantly older than the LRF/LRC group (642 ± 225 vs. 536 ± 234 days, Mann-Whitney test, p-value = 0.03).

Significant effects of Genotype, Age and Farm on the response variable were evidenced by models and were accounted to provide the most accurate estimate of the marginal effect of the genotype (Supplementary Table 5). The optimal model for predicting chest circumference, based on the lowest Akaike Information Criterion (AIC 657.4), with Bayesian information criterion (BIC 679.5), log-likelihood (−319.7), deviance (639.4), and 77 residual degrees of freedom, balances simplicity and fit. It uses a Gaussian family with an identity link and includes Genotype, Age, and a random effect for Farm. Random effects analysis showed farm-level intercept variance of 80.63 (SD =8.98), indicating variability in chest circumference across farms. Fixed effects revealed that the LRS genotype was associated with a significant reduction in chest circumference (−13.10 ± 1.75 cm, p-value = 6.94 × 10^–14^), independent of age. Age, modelled by natural splines, showed a non-linear effect, with all spline terms significantly positive (*p* < 2 × 10^–16^), and the largest effect at the fourth spline (147.355). In conclusion, this model highlights the significant impact of the mutation on chest circumference, with genotype and age as key factors, and a random farm effect incorporated. Consequently, the model indicates BLIRD is associated with reduced chest circumference. The estimated marginal means (EMMs) for each genotype is visualised in Figure 2D, and various chosen age points are provided in Supplementary Table 6. Notably, for chest circumference, LRS animals in the model would have a chest circumference at 480 days of 164 cm VS. 177 cm for LRF/LRC. The calculated weight around breeding time (which corresponds to 356 kg VS. 441 kg), represents only 50% of the desired mature weight of 700 kg, which may explain the delays in breeding.

The analysis of variables in models shows significant genotype effects on several variables, including chest circumference, Haemoglobin, lymphocytes, monocytes and eosinophils. In particular, chest circumference showed a strong Genotype effect (chi-squared =68.61, *p* < 1.20 × 10^–16^) and a significant non-linear Age effect (chi-squared =258.91, *p* < 6.72 × 10^–54^). However, the genotype by age interaction for chest circumference was not significant (p-value = 0.07). Genotype was also a significant (*p* < 0.05) factor for Haemoglobin, lymphocytes, monocytes and eosinophils (Supplementary Table 7). In addition, Age had a significant effect on urea (chi-squared =26.56, p-value = 6.94 × 10^−5^).

### BLIRD homozygote carriers have a severely perturbed haematological profile with a severe lymphocytosis

The cattle examined came from herds that were free of regulated diseases, such as tuberculosis, brucellosis and enzootic bovine leukosis. BVDV and BoHV-1 molecular and serological detections were negative for both cases and LRF/LRC, except in a few vaccinated herds to BVDV, supporting the absence of active and ancient infection. Similarly, *Mycobacterium avium* subsp *paratuberculosis* faecal detection was all negative, and they were serologically negative for *M. paratuberculosis* antibodies, excluding a major role of these infections in the clinical findings.

To assess the consequence of BLIRD on inflammatory parameters, systemic biomarkers in blood and blood biochemistry were evaluated between genotype groups (Supplementary Table 3 and 7). Urea levels were not different between the LRF/LRC and LRS groups (3.6 ± 1.4 vs. 3.0 ± 1.5 mmol/L respectively, Mann-Whitney, p-value = 0,14). Similarly, aspartate aminotransferase (AST) values did not vary according to the group (118 ± 127 vs. 104 ± 40, Mann-Whitney, p-value = 1). Plasma total proteins (73.5 ± 8.7 vs. 75.3 ± 6.5 g/L, Mann-Whitney, p-value = 0,11), and albumin concentrations (33.6 ± 5.7 vs. 31.3 ± 3.4 g/L p-value = 0,13) were not different on average, while globulinemia was significantly higher in the group (39.9 ± 4.0 vs. 44.0 ± 4.5 g/L, Mann-Whitney test, p-value = 1.42 × 10^−3^).

**Figure 3.**
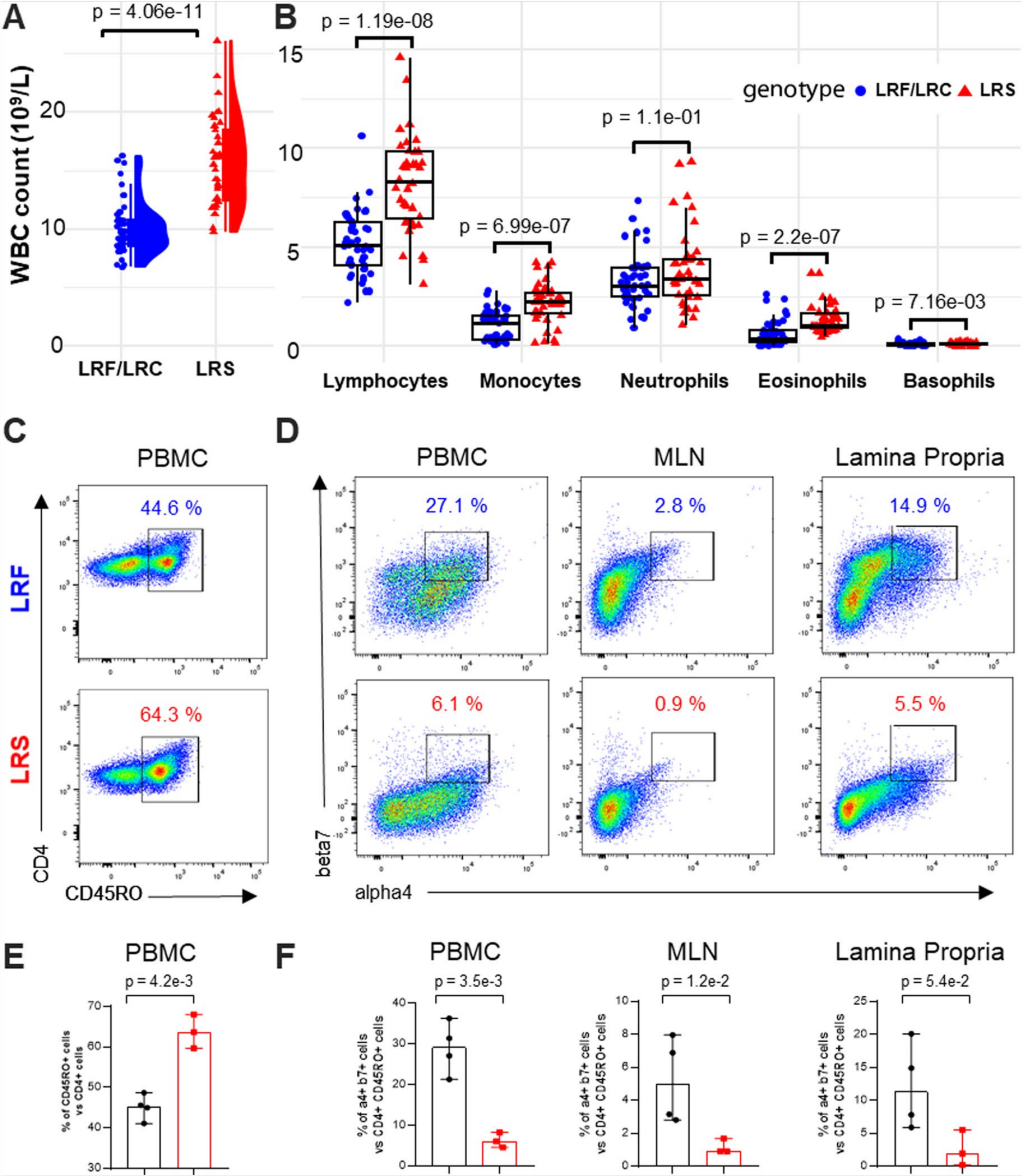
Blood count and tissue distribution of LPAM1^pos^ CD4 T cells in LRS and LRF/LRC animals. (A) White blood cell count (in 10^9^ cells/L) in LRS and LRF/LRC animals. (B) Blood cell counts (in 10^9^ cells/L) of lymphocytes, monocytes, neutrophils, eosinophils, and basophils in LRS (n = 36) and LRF/LRC (n = 40) animals. (C) Representative flow cytometry plots of CD45RO^+^ cells among CD4^+^ T lymphocytes in peripheral blood (PBMCs) from LRS (n = 3) and LRF/LRC (n = 4) animals. (D) Representative flow cytometry plots of α4^+^β7^+^ cells among CD45RO^+^ CD4^+^ T cells in peripheral blood (PBMCs), mesenteric lymph node (mLN) and jejunal lamina propria (jLP) from LRS (n = 3) and LRF/LRC (n = 4) animals. (E) Quantification of CD45RO^+^ cells among CD4^+^ T lymphocytes in peripheral blood (PBMCs) from LRS (n = 3) and LRF/LRC (n = 4) animals. (F) Quantification of α4^+^β7^+^ cells among CD45RO^+^ CD4^+^ T cells in peripheral blood (PBMCs), mesenteric lymph node (mLN) and jejunal lamina propria (jLP) from LRS (n = 3) and LRF/LRC (n = 4) animals.

In contrast to biochemistry, complete blood count (CBC) exhibits variation according to the genetic group. Mean white blood cell count (WBC) was 150% higher (15.7 ± 3.7 vs. 10.2 ± 2.3 × 10^9^, Mann-Whitney test, p-value = 4,06 × 10^−11^) in the LRS group (Figure 3A), and a significant difference was observed for lymphocyte count, (8.2 ± 2.4 x 10^9^ vs. 5.2 ± 1.6 x 10^9^, Mann-Whitney test, p-value = 1.19 × 10^−8^) (Figure 3B). A markedly elevated population of eosinophils is present in the LRS in comparison to the LRF/LRC group (0.6 ± 0.6 vs. 1.4 ± 0.7, Mann-Whitney, p-value = 2.20 × 10^−7^). Similarly, monocyte count (2.2 ± 1.1 vs. 1.0 ± 0.7, Mann-Whitney, p-value = 7.0 × 10^−7^) and basophils (0.11 ± 0.06 vs. 0.08 ± 0.08, Mann-Whitney, p-value = 7.16 × 10^−3^) were also higher in the former contributing to the CBC difference. In contrast, the neutrophil count (3.9 ± 1.9 vs. 3.3 ± 1.4 × 10^9^ cell/L, Mann-Whitney test, p-value = 0,11) and the red blood cell count (RBC, 7.2 ± 1.0 vs. 7.7 ± 1.0 × 10^12^ cell/L, Mann-Whitney test, p-value = 0,03), were within the reference ranges and not different between the groups. Haemoglobin concentration (Hb, 10.4 ± 1.4 vs. 11.1 ± 1.3 g/L, p-value = 5,28 × 10^−3^), was significantly lower in the LRS group. A comprehensive overview of the biochemical and haematologic findings between LRF/LRC and LRS is provided in Supplementary Table 7.

We subsequently examine the lymphocyte population to look for differences in their distribution across blood and digestive tissues, and to see how they can explain the difference in lymphocyte count between the two groups. LPAM-1 (integrin α4β7 dimer) has been shown to mediate the homing of memory (recognized as CD45RO^pos^ in cattle) CD4 T lymphocytes into the gut area (Wagner et al., 1996; Williams and Butcher 1997). Naive T cells exhibit low levels of expression of CD45RO, which increase upon activation and differentiation into effector and memory T cells (Kilshaw and Murant 1991). For that reason, we examined the integrin α4 and β7 expression on CD45RO^pos^ CD4^pos^ T cells by flow cytometry in the blood of a small group of animals (three LRS and four LRF/LRC). Figure 3C and E show that the LRS have a larger memory cell population among CD4 T lymphocytes than the LRF/LRC animals’ group (63.8% vs. 45,1%, t-test, p-value = 4.2 × 10^-^³). Furthermore, the proportion of cells expressing integrin α4 and β7 among CD45RO^+^CD4^+^ T cells was reduced in LRS compared to LRF/LRC (Figure 3D), either in the blood (6.3% vs. 29.0%, t-test, p-value = 3.5 × 10^-^³), in the draining mesenteric lymph node (1.2% vs. 5.2%, t-test, p-value = 1.2 × 10^−2^), and in the intestine (jejunal lamina propria) of the LRS, which contained 2.7-fold fewer LPAM-1^+^ lymphocytes compared to LRF/LRC, with a trend towards a difference (2.6% vs. 12.2%, t-test, p-value = 5.4 × 10^−2^). The proportion is shown in Figure 3F.

These findings support the idea that BLIRD cattle have an impaired retention of CD4 T lymphocytes in the lamina propria (LP), and an observation behind the BLIRD designation of the genetic disorder, with fewer α4β7 ^+^ T cells in mesenteric lymph nodes (mLNs), and submucosal space (LP), despite overabundant CD45RO^pos^ CD4^pos^ T lymphocytes in the blood.

### Histopathological findings

**Figure 4.**
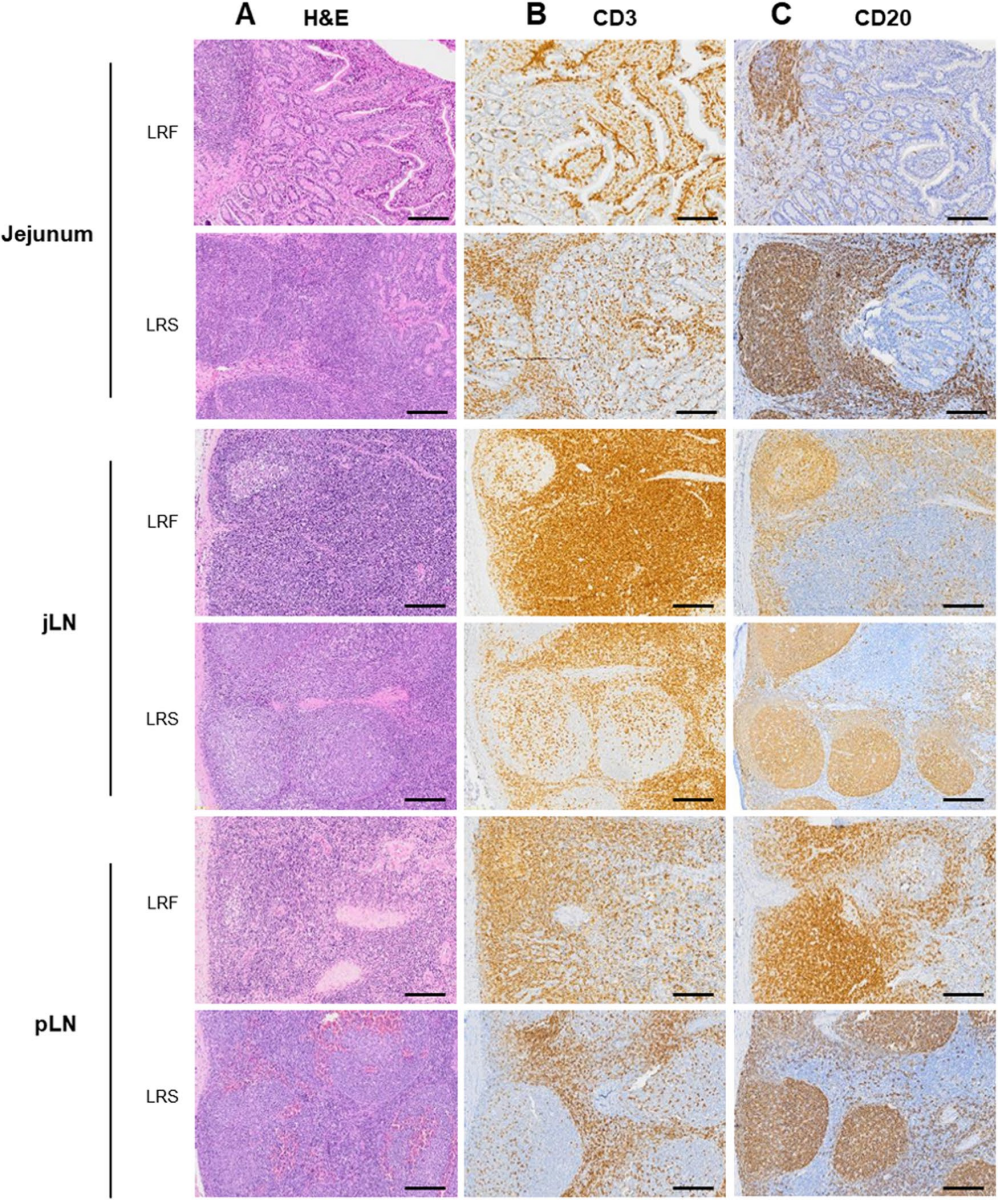
Histopathology of the jejunum, jejunal lymph node (jLN) and peripheral lymph nodes (pLN) of a LRS and a LRF/LRC animal. (A) Haematoxylin and eosin staining of jejunum, jejunal lymph node and peripheral lymph node tissues of a LRS and a LRF/LRC. (B) CD3 (T lymphocyte marker) and (C) CD20 (B lymphocyte marker) immunostaining of the same tissues of a LRS and a LRF/LRC.

At necropsy, LRS weighed 300, 387 and 550 kg. All examined organs were within normal limits with no evident abnormalities that could explain the animal’s suboptimal health conditions or premature demise. Histologically, mutated animals presented moderate to marked cortical follicular lymphoid hyperplasia in peripheral (prescapular) lymph nodes (3/3) and intestinal Peyer’s patches (3/3) (Figure 4A, Hemalun&eosin staining). Paracortical areas were within normal limits or mildly reduced in size due to expansive areas of follicular hyperplasia in mutated animals. Other lymphonodal follicular changes were observed, including intra-follicular haemorrhages, hyalinosis and dystrophic mineralisation. Intestinal mucosa, as well as other tissues, were within normal limits in comparison with tissue from LRF/LRC subjects. Immunohistochemistry confirmed the abundance of CD20^pos^ lymphoid cells within affected lymph nodes and Peyer’s patches, with prominent follicular organisation (Figure 4B and C). CD20^pos^ cells frequently showed a moderate (2/3) to marked (1/3) increase in number and frequency within intestinal lamina propria in subjects. In contrast, CD3^pos^ lymphoid cells were present, heterogeneously distributed throughout paracortical areas of the lymph nodes from subjects. They were less frequent and mildly reduced in density in the jejunal lymph node (3/3) and prescapular lymph node (2/3). In the intestinal mucosa, CD3^pos^ cells were observed expanding heterogeneously in the deep lamina propria of subjects (3/3), in contrast with tissues from the LRF/LRC where CD3^pos^ cells were mostly observed within superficial villous interstitium and epithelium. Overall histological data suggest intestinal, peripheral and loco-regional lympho-nodal B-cell lymphoid hyperplasia. No significant changes in T-cell density were observed but substantial topographical changes were noted.

### BLIRD affects the faecal microbiota in LRS cows

**Figure 5.**
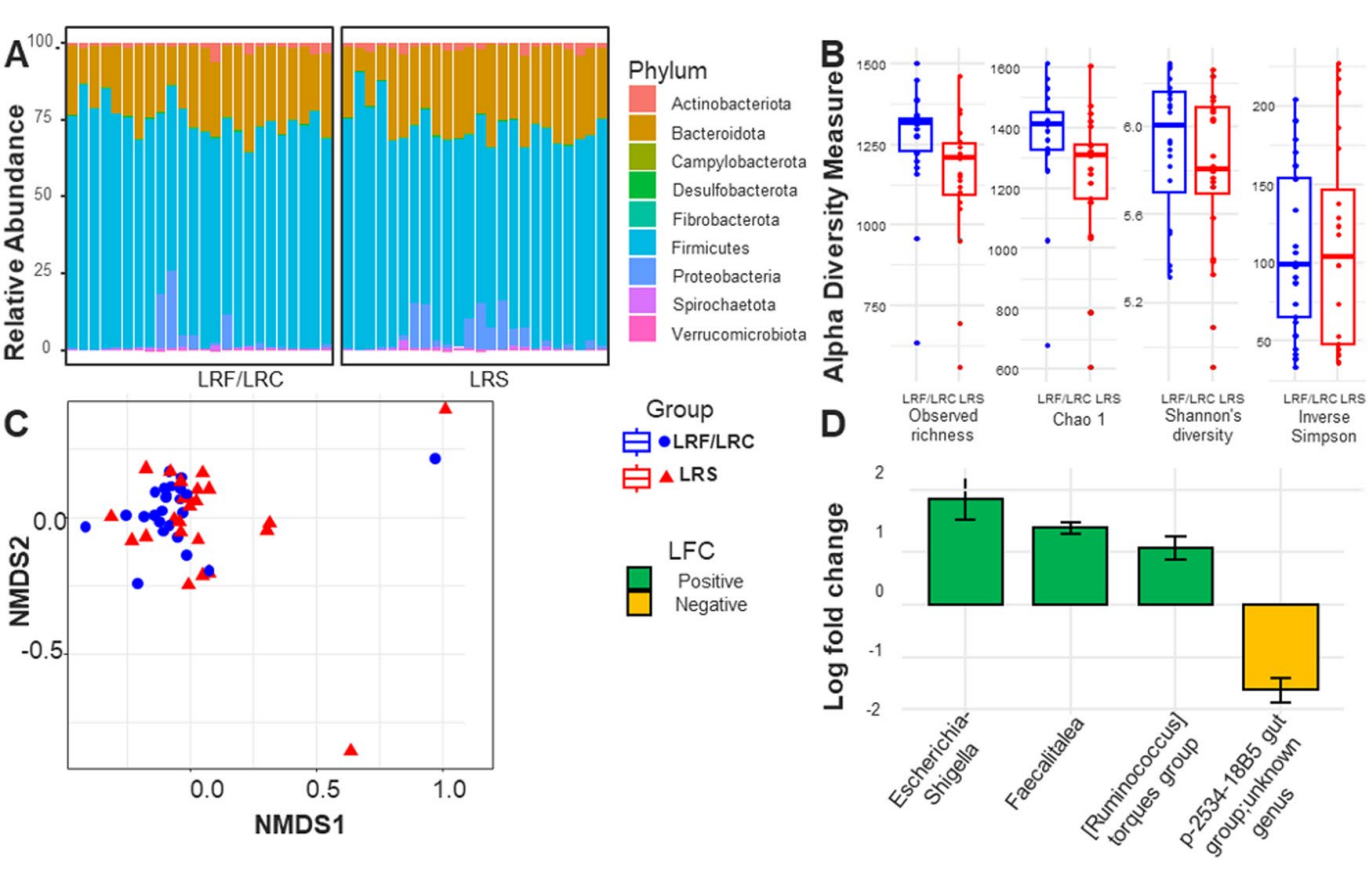
Composition of the faecal microbiome of 21 couples of LRS and LRF/LRC animals in different herds. (A) Relative abundance of phyla between LRF/LRC and LRS groups. (B) Alpha diversity measures, including observed richness, Chao1, Shannon’s diversity index, and inverse Simpson index, comparing LRF/LRC and LRS groups. (C) Beta diversity, represented by Bray-Curtis dissimilarity distances between LRF/LRC (blue rounds) and LRS (red triangles) animals. (D) Log fold change at the Genus level comparing LRF/LRC and LRS groups, showing only significant differences.

The systemic and local (intestinal) immune system is exposed to and interacts closely with the microbiota in the digestive tract as the main trigger of its development (Molloy et al. 2012). In line with the evident changes in the adaptive compartment of the digestive tract, we investigated differences in the faecal microbiota composition. The microbiota composition of the faeces was examined in 21 couples of LRS and LRF/LRC in different commercial farms and represented on a phylum scale (Figure 5A). We identified notable differences in the taxonomic composition between the two groups, with some bacterial populations that are in greater proportions in the cases. Indeed, as Figure 5B illustrates, there are substantial disparities in alpha-diversity between the two groups (p-value <0.05), as evidenced by all indicators including observed richness, Chao1, and Shannon’s, except for the inverse Simpson index, indicating a disruption in microbial biodiversity in the group. Figure 5C illustrates beta-diversity through Bray-Curti’s dissimilarity distances, showing no discernible separation in the microbiota composition between the LRS and LRF/LRC animals. We next interrogated the difference at the Genus level and found significant differences in microbial abundance for *Escherichia/Shigella* (2.00 log-fold change), *Faecalitalea* (1.45 log-fold change), *[Ruminococcus] torques group* (1.06 log-fold change) and unknown genus from *p-2534-18B5 gut group* (−1.63 log-fold change) illustrated in Figure 5D. Our findings show a substantial alteration in the microbiota composition in the LRS compared to their LRF counterparts.

### Default stature and few blood parameters typify carrier from non-carrier status

**Figure 6.**
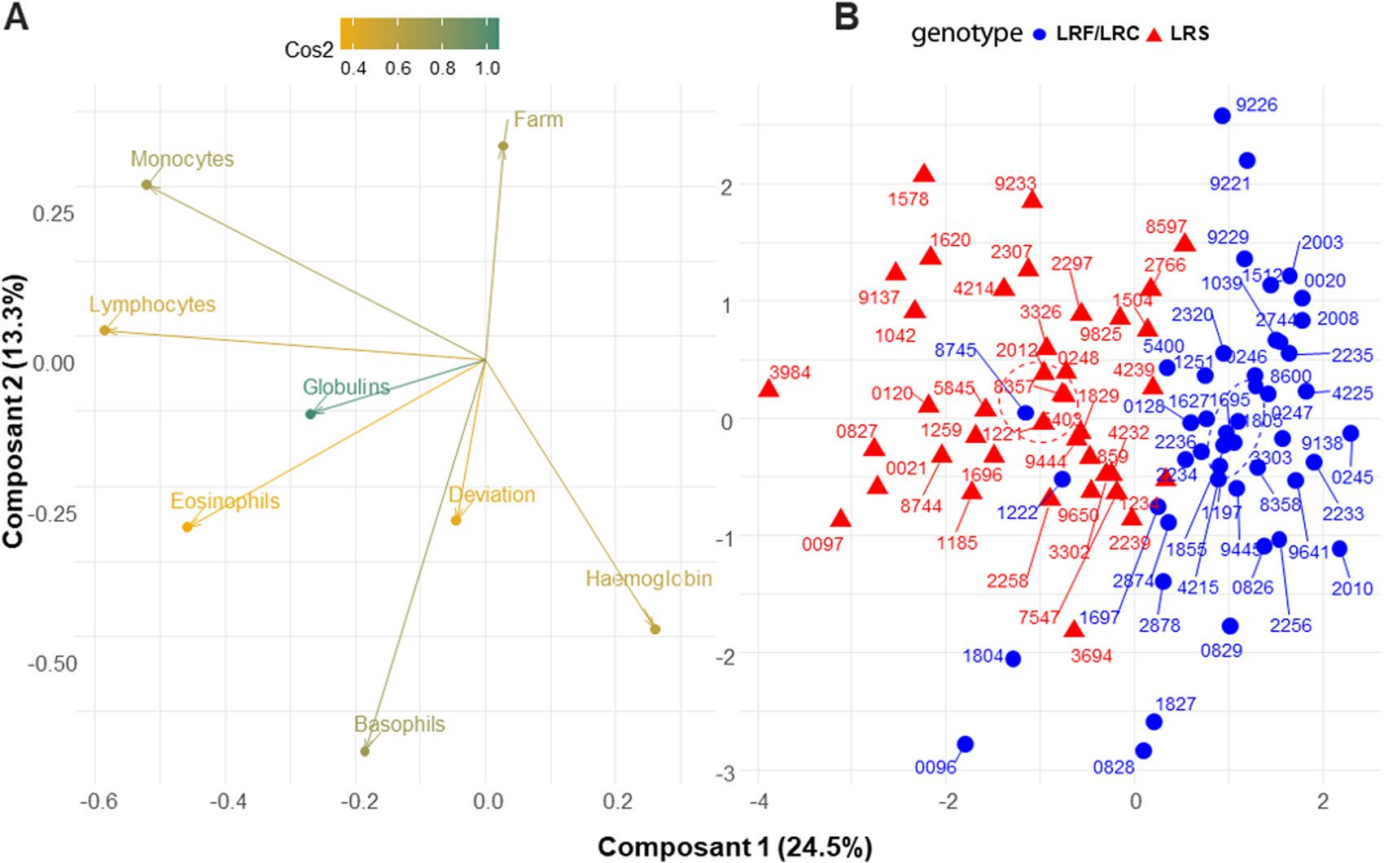
Sparse partial least squares-discriminant analysis (PLS-DA) on clinical and biological parameters for identification of LRS animals. (A) Representation of variable contributions, with colours indicating their relative importance (cos2). (B) sPLS-DA plot for individuals with labels and ellipses with shapes with colour coding based on genetic status with LRS (red triangle), and LRF/LRC (blue round).

The subsequent inquiry sought to ascertain the ease with which homozygote carriers could be identified within the cattle population. Utilising multifactorial analysis (MFA) and principal component analysis (PCA), we examined the influence of genotype and the values of associated uncorrelated biological variables on classification. PCA biplots demonstrated that individuals with identical genotypes formed distinct clusters, thereby substantiating the robustness of genotype-related patterns. Hierarchical clustering further demonstrated that individuals were grouped according to Farm, indicating a potential effect of the environment on the classification process. The sPLS-DA assessment revealed that Principal Component 1 (PC1), accounting for 24.5% of the total variance, is predominantly comprised of lymphocytes, with monocytes, eosinophils, basophils, and globulins exhibiting a negative correlation with the LRS status (increased). Principal Component 2 (PC2) is predominantly composed of farm and Haemoglobin, which is inversely correlated with the LRF/LRC group (Figure 6A). The sPLS-DA components presented in Figure 6B showed a distinct separation of the LRS and LRF/LRC samples. To conclude, the sPLS-DA results demonstrated that immune-related markers (e.g. lymphocytes, eosinophils monocytes, and basophils) played pivotal roles in both components, with Farm emerging as a significant secondly positive contributor. These findings underscore the intricate relationships between immune markers and genotype, reflecting the multifaceted biological processes and environmental conditions that underlie the phenotype data. This integrative analysis demonstrates that only a limited set of variables, including lymphocyte count and growth retardation, are effective in differentiating LRS from their LRF/LRC.

## Discussion

Our study aim was to gain insights into the clinical manifestations and underlying mechanisms of BLIRD, a recessive genetic disorder (ITGB7 p.G375S) recently discovered in Holstein cattle (Besnard et al. 2024). We provide essential information for the future management of this genetic disorder in the affected dairy cattle population and interesting observations on the immune system defects associated with a dysfunctional variant of integrin β7.

Growth retardation is common in cattle and identification of the underlying cause is a major challenge for cattle practitioners, leading to suboptimal performance and reduced longevity in breeding programs (Heinrichs et al. 2017). Poor condition in animals can be caused by malnutrition, parasitism, chronic infections, genetic disorders, and environmental factors like nutrition, housing, and infectious disease exposure. In this study, animals with the mutation showed reduced longevity compared to LRF/LRC. Factors contributing to this include premature death, progressive emaciation, poor condition, and sudden death at a time of expected exponential growth. Breeders did not detect any specific illness, and the attempted treatments did not resolve the issue. Additionally, low development and insufficient stature led to early culling.

The optimal age for AI in dairy heifers is approximately 15 months, corresponding to around 55% of their mature weight (Smith 2007) to ensure they attain sexual maturity and achieve optimal fertility (Le Cozler et al. 2008). Delaying AI beyond recommended timeframes leads to reduced reproductive efficiency, prolonged inter-calving intervals, reduced lifetime productivity, health complications, and metabolic disorders, affecting dairy production sustainability and non-productive life (Wathes et al. 2014). Third, lower lactation performances for primiparous cows are another reason for reduced productive life by early culling (De Vries and Marcondes 2020).

The study found no significant difference in milk quality assessed by somatic cell concentrations, suggesting no predisposition to common infections like mammary infection and mastitis. The statistical power of the analyses is limited by the low number of homozygotes available and the magnitude of the effect may be underestimated. Clinical descriptions and quantitative parameters can help detect suspected cases without genetic testing.

In our study, variants are smaller and in poorer condition than matched control. To reduce the impact of confounding variables, the age difference in each couple of LRF/LRC and LRS in the same herd was maintained at a minimum, typically less than a month in most cases. However, due to their shorter stature, homozygous carriers of the point mutation were frequently raised with younger stock. This resulted in heifers of similar age being located in different areas or eventually absent in small-size herds, thus unavailable for pairing. This discrepancy in age between LRS and LRF/LRC can be attributed to LRS growth retardation. The statistical approach adopted effectively reduced the confounding effect of age, thereby providing a clearer understanding of the genotype effect on several measures, including chest circumference.

Thus, the field analyses have shown how the BLIRD affects the longevity of Holstein dairy cows, with growth retardation and inappropriateness to breeding in homozygous animals under most herd conditions. No impaired, nor favourable traits were observed in the heterozygotes compared to matched wild-type suggesting no advantage in heterozygosity. Given the detrimental effects of the variant on dairy cow health and welfare, carriers should be excluded from breeding. Digital breeding management can aid early detection and guide informed mating decisions to gradually eliminate the mutation from the population.

The integrin β7 variant chain, a subunit of the integrin α4β7 LPAM-1 dimer, is crucial for immune cell distribution under homeostatic conditions. The mutation could have impaired an animal’s ability to respond to digestive infections by pathogens like viruses, bacteria, or helminths. However, no digestive signs or lesions were found in homozygous carriers of the variant. A few LRS were necropsied, so that an observational bias in the case recruitment cannot be excluded.

The homing of CD4 T lymphocytes to gut-associated lymphoid tissues (GALT) is best described among α4β7 LPAM-1-expressing cells (Besnard et al. 2024). In LRS cattle, the point mutation likely disrupts dimer expression, impairing lymphocyte diapedesis and reducing memory CD4 T cells in the lamina propria, suggesting compromised gut and systemic immune memory. Additionally, LRS exhibit significantly higher circulating leukocytes, including lymphocytes, eosinophils, and monocytes, compared to control LRF/LRC animals.

Several studies have described the expression of integrin β7 at the cell membrane of these cells, indicating that this adhesion molecule may be necessary for their relocation and full activity. In integrin β7 knockout mice (Wagner et al., 1996), or following treatment with Vedolizumab, a monoclonal antibody that blocks the interaction between LPAM-1 and MadCAM-1 (Canales-Herrerias et al. 2024); lymphocyte migration to the GALT is impaired, potentially disrupting intestinal homeostasis and immune tolerance. Consistent with these findings, our examination reveals a deficit in LPAM-1 expression in LRS and precisely on activated/memory CD4+ T cells. However, unlike these studies, we observed an accumulation of those lymphocytes in peripheral blood, which may be explained by the inability of these cells to migrate towards endothelial cells. Furthermore, the lack of migration of various lymphocyte subsets could hinder proper immune surveillance, increasing susceptibility to parasitic infections, as demonstrated in mice infected with *Trichinella spiralis* (Song et al. 2019). Additionally, in cases of BLIRD, a pronounced lymphocytosis was observed, alongside eosinophilia and monocytosis. Lymphocytosis in cattle is typically associated with chronic infections or bovine leukaemia virus (Abramowicz et al. 2019), yet all studied herds were free of these. Notably, the ITGB7 p.G375S mutation appears to induce lymphocytosis independently, suggesting a chronic immune activation state with accumulation of memory CD4 T cells in blood and follicular hyperplasia in lymph nodes.

This is consistent with findings from other studies, which have shown that mutations affecting immune system function, such as those in integrins, can lead to persistent lymphocyte proliferation (Moser et al. 2009; Calderwood et al. 2013).

Eosinophilia develops due to allergy, inflammatory responses and neoplasm (Ramirez et al. 2018; Morales-Camacho et al., 2024 discard). It’s also associated with parasitic infections (Claerebout and Vercruysse 2000) such as the response to helminths, particularly by gastrointestinal nematodes (e.g. *Ostertagia spp.*, *Cooperia spp.*) and lungworms (e.g. *Dictyocaulus viviparus),* no preferential excretion in LRS was shown in our study. This may be related to low or no exposure due to no grazing in most farms. Parasitism is not a sound explanation for ill-thrift and growth retardation. Hypersensitivity reactions or fungal infections can trigger a similar immune response, particularly in chronic or granulomatous inflammation, in which eosinophils contribute to tissue repair (Coden and Berdnikovs 2020). Additionally, autoimmune diseases and certain neoplasms can cause persistent eosinophilia due to abnormal immune system activation and cellular proliferation (Diny et al. 2017; Shomali and Gotlib 2024) but no evidence was collected to support this possibility.

Most LRS showed low haemoglobin concentration, a multifactorial condition often caused by nutritional deficiencies (Suttle 1999), chronic inflammation, or parasitic infections (Constable et al. 2017), none of which were present in these animals.

Our study found an increase in plasma globulins, a common symptom of chronic immune activation in cattle with persistent infections or ageing. This could be linked to lympho-nodal follicular hyperplasia, a reactive benign proliferation affecting lymphoid cells, which is associated with adaptive B-cell immune tissue in response to infections, immune-mediated disorders, and certain malignancies. The continuous antigenic challenge comes from the feed and gut microbiota that play a pivotal role in shaping the immune system, particularly in cattle, where the rumen and intestines are home to large and diverse microbial communities (Keum et al. 2024). Diet, environmental conditions, and genetic factors all contribute to the composition of the gut microbiota, which in turn affects the host’s immune system (Jami and Mizrahi 2012). Some authors (Cendron et al. 2020) examined the faecal microbiomes of Holstein-Friesian and Simmental heifers and lactating cows, identifying significant differences in microbial composition between the two groups within the same herd. The predominant bacterial phyla were *Firmicutes*, *Bacteroidetes*, *Actinobacteria*, and *Proteobacteria*, with variations in bacterial families and genera observed between heifers and cows, suggesting that the faecal microbiome can be influenced by management conditions. While age-related variation could introduce bias in our study, the mutated animals, although slightly older, were on the same diet and at the same physiological stage as their control, minimising this factor. In case of immune dysfunction, disruptions in the gut microbiota may exacerbate immune system imbalance (Jiao et al. 2020), further compromising health and growth (Xu et al. 2021), such as those with the integrin β7 mutation. Indeed, examination of the gut microbiota’s influence on immune system function is particularly relevant in this context, as impaired immune cell migration to the GALT could lead to an altered microbial environment of the digestive tract and reciprocally. This disruption may affect the balance between beneficial and harmful microbiota, further impairing digestion, nutrient absorption, and immune surveillance. In contrast to what has been described in knockout mice where a few Operational Taxonomic Units (OTU) were a little more abundant (Babbar et al. 2019), we found a clear difference in the microbiota composition between LRS and their within-farm matched LRF/LRC controls. First, α and β diversities were affected, and the abundances of several bacteria phyla and genera were significantly different between the two variant categories. Interestingly, several of these bacteria were previously associated with pathological conditions in Bovidae. The *Ruminococcaceae NK4A214 group* was more abundant in the LRS group as similarly described (Ma et al. 2020), studying the gastrointestinal tract bacterial communities in normal and growth-retarded yaks. The gut microbiota of yaks afflicted by diarrhoea was examined (Wu et al. 2022), unveiling that the microbial composition exhibited substantial alterations in the diarrhoea group compared to healthy LRF/LRC. As in the aforementioned study, we observed a decrease in alpha diversity in the LRS group, alongside an increase in the genus *Escherichia-Shigella*. Similarly, some authors found a significant reduction in microbial diversity in diarrhoeic animals, along with alterations in the composition of the gut microbiota in diarrhoeic Père David’s deer (Zhen et al. 2022). Additionally, alteration in the gut microbiota could lead to nutritional deficiencies and further compromise the animal’s feeding behaviour, growth and productivity (Clemmons et al. 2019; Welch et al. 2022).

Poor growth in animals may be due to metabolic issues, such as inadequate nutrient assimilation, which can be complicated by the inability to effectively utilise nutrients. Abnormal absorption in the intestine is plausible, but the lack of structural changes and chronic inflammation lowers the likelihood. Immune dysfunction can affect nutrient uptake and utilisation, causing weight loss and reduced body condition (Ingvartsen and Moyes 2013). Moreover, some integrin beta7^pos^ T cells regulate the digestive epithelium for nutrient absorption by mitigating GLP-1 availability, rendering *ITGB7* knockout mice metabolically hyperactive (He et al. 2019). These mechanisms have not yet been described or even studied in the bovine species.

The study on BLIRD genetic disorder in Holstein cattle offers valuable insights into immune defects. However, it has limitations and potential biases. Farmers’ willingness to participate in the survey was uncontrollable, and the cattle were recruited from a pool of genotyped cattle in France. Advanced technical and management practices may influence the observed outcomes. Out of 40 LRS, only 27 pairs of matched case LRF/LRC were formed due to the inability to find an appropriate LRF/LRC within the herd. Necropsied animals were chosen as representative of most clinical presentations, confirming the observations are typical of BLIRD.

BLIRD represents a novel genetic disorder in Holstein cattle, with significant implications for animal health and breeding practices. The present study proposes a novel approach to the investigation of the function of integrin β7, employing an animal model that allows for the comparison of results across cattle and other species. Furthermore, α4β7-MAdCAM-1 interaction is also a therapeutic target in inflammatory bowel diseases (IBD) in humans and developing more effective therapeutic agents and broader applications beyond is needed. Comparative and translational immunology between species may help understand integrin β7 function in the development and homeostasis of the immune system.

## Supplementary Material

Dutheil_suppl_data.xlsx

## Data Availability

The raw sequences related to 16S rRNA gene analysis are available in the National Centre for Biotechnology Information Sequence Red Archive under the Bio Project accession number PRJNA1297424 (https://www.ncbi.nlm.nih.gov/bioproject/PRJNA1297424/).
